# Design and modeling of pulsed-laser three-dimensional imaging system inspired by compound and human hybrid eye

**DOI:** 10.1038/s41598-018-35098-9

**Published:** 2018-11-21

**Authors:** Yang Cheng, Jie Cao, Fanghua Zhang, Qun Hao

**Affiliations:** Beijing Key Laboratory for Precision Optoelectronic Measurement Instrument and Technology, School of Optics and Photonics, Beijing Institute of Technology, Beijing, 100081 China

## Abstract

A pulsed-laser three-dimensional imaging system inspired by compound and human hybrid eye is proposed. A diffractive optical element is used to enlarge field of view (FOV) of transmitting system and a receiving system consisting of a non-uniform microlens array, an aperture array, and an avalanche photodiode array is designed. The non-uniform microlens array is arranged on a curved surface to mimic large FOV feature of the compound eye. Meanwhile, the non-uniform microlens array is modeled to mimic space-variant resolution property of the human eye. On the basis of the proposed system, some simulation experiments are carried out. Results show that the entire FOV is up to 52°, and the resolution is 30 × 18. The proposed system has a high resolution in the center FOV and a low resolution in the peripheral FOV. The rotation and scaling invariances of the human eye are verified on the proposed system. The signal-to-noise ratio (SNR) increases with the increase in the number of rings and the maximum SNR locates at the outmost periphery area. This work is beneficial to the design of the pulsed laser three-dimensional imaging system with large FOV, high speed, and high resolution.

## Introduction

In recent years, pulsed-laser three-dimensional (3D) imaging has become as an active imaging technology and has received much attention due to its simplicity in principle, ability of anti-interference, and long imaging distance range^1,2^. Different from two-dimension planar imaging, pulsed-laser 3D imaging is slightly affected by target illumination and background reflectance, and it can obtain depth information of targets^3^. Therefore, this imaging technology is widely used in various applications, including target recognition^4^, robotics^5^, terrain visualization^6^, medical diagnostics^7^, and vehicle navigation^8^. A pulsed-laser 3D imaging system with a large field of view (FOV), high imaging speed and high imaging resolution is critical and exhibits a huge potential in many applications, such as industry, medical and military areas^7,9,10^. To the best of our knowledge, the imaging principle divides the pulsed-laser 3D imaging system into two categories: scanning and non-scanning types^11^. For scanning pulsed-laser 3D imaging system (e.g., MEMS, scanning mirror, galvanometer scanner, and Risley prism pair), the FOV largely depends on scan range of scanning components and the capability to obtain a high resolution image relies largely on the high-resolution scanning angle and a large number of scanning steps of the scanning components, which results in a high time consumption^12–16^. Therefore, it suffers from a low imaging speed that limits its imaging efficiency. Moreover, mechanical robustness is a substantially challenging issue for this type of pulsed-laser 3D imaging system. Unlike scanning pulsed-laser 3D imaging system, non-scanning pulsed-laser 3D imaging system can obtain images with only a single laser pulse in a short period. The FOV of non-scanning pulsed-laser3D imaging system mainly depends on receiving optical system. Imaging with a FOV over 90° has been achieved with fisheye lenses, catadioptric lens, and rotating cameras^17,18^. However, the imaging system requires bulky and costly multiple-lenses and stringent alignment. Moreover, it suffers from a severe aberration, which will deteriorate the imaging quality^19^. The non-scanning pulsed-laser 3D imaging system permits a high imaging speed in the absence of the scanning component, but the image resolution depends on the pixel number of a detector array. A large pixel number of the detector array is necessary to obtain an image with high resolution^20–25^. Actually, high resolution within the entire FOV is unnecessary, i.e., the resolution through the entire FOV does not have to be the same because high resolution image requires large data bandwidth, which limits the image speed. The analysis above shows that a tradeoff exists among large FOV, high speed, and high resolution; they cannot be satisfied simultaneously by the existing pulsed-laser 3D imaging systems^26^.

Compound and human eyes are two exquisite and outstanding animal eyes created by nature^27^. On one hand, the compound eye has a remarkable feature of large FOV because its ommatidia are distributed on a hemispherical curved surface^28,29^. Each ommatidium only transfers a small part of the FOV and the final FOV is the cumulation result of all ommatidium. On the other hand, one unique merit of the human eye is the space-variant resolution property, i.e., high resolution in the center of the FOV and low resolution in the peripheral of the FOV^30,31^. Benefiting from this property, it can compress image information in the periphery of the FOV, i.e., the uninterested area meanwhile maintains the image information in the central of the FOV, i.e., the interested area clear^32,33^. Hence, the space-variant resolution property improves imaging speed and possess high resolution in the interested area. The space-variant resolution property demonstrates that the relationship between the retina and visual cortex is subjected to an approximate logarithmic-polar transformation (LPT)^34^. It can convert a Cartesian image of the retina to a LPT image of the visual cortex. The LPT of the human eye has two remarkable properties, i.e., rotation and scaling invariance, which are beneficial in further reducing redundant data and compressing the data^35^. It is particularly helpful for pattern recognition, motion estimation and object tracking. To the best of our knowledge, there have been a few previous studies on the large FOV feature of the compound eye^36–38^ and the space-variant resolution property of the human eye^39–41^, respectively. However, the hybrid of the compound and human eyes has not yet been studied. Inspired by bionic technology, our group previously proposed an imaging system combining of these two eyes, but it is a passive imaging system and belongs to two-dimensional planar imaging^30,42^. Unlike our previous studies, we study a pulsed-laser 3D imaging method combined the large FOV feature of the compound eye and the space-variant resolution property of the human eye simultaneously.

To obtain the large FOV, high imaging speed, and high resolution, a novel imaging method inspired the compound eye and the human eye for the pulsed-laser 3D imaging system is proposed. A receiving system containing several non-uniform microlenses is designed to mimic the space-variant property of the human eye. The aim of this design is to achieve high resolution in the center of the FOV meanwhile compress the image information in the periphery of the FOV for improving imaging speed. Moreover, these non-uniform microlenses are distributed on a curved surface to imitate the large FOV feature of the compound eye.

## Methods

### Principle

**Figure 1:**
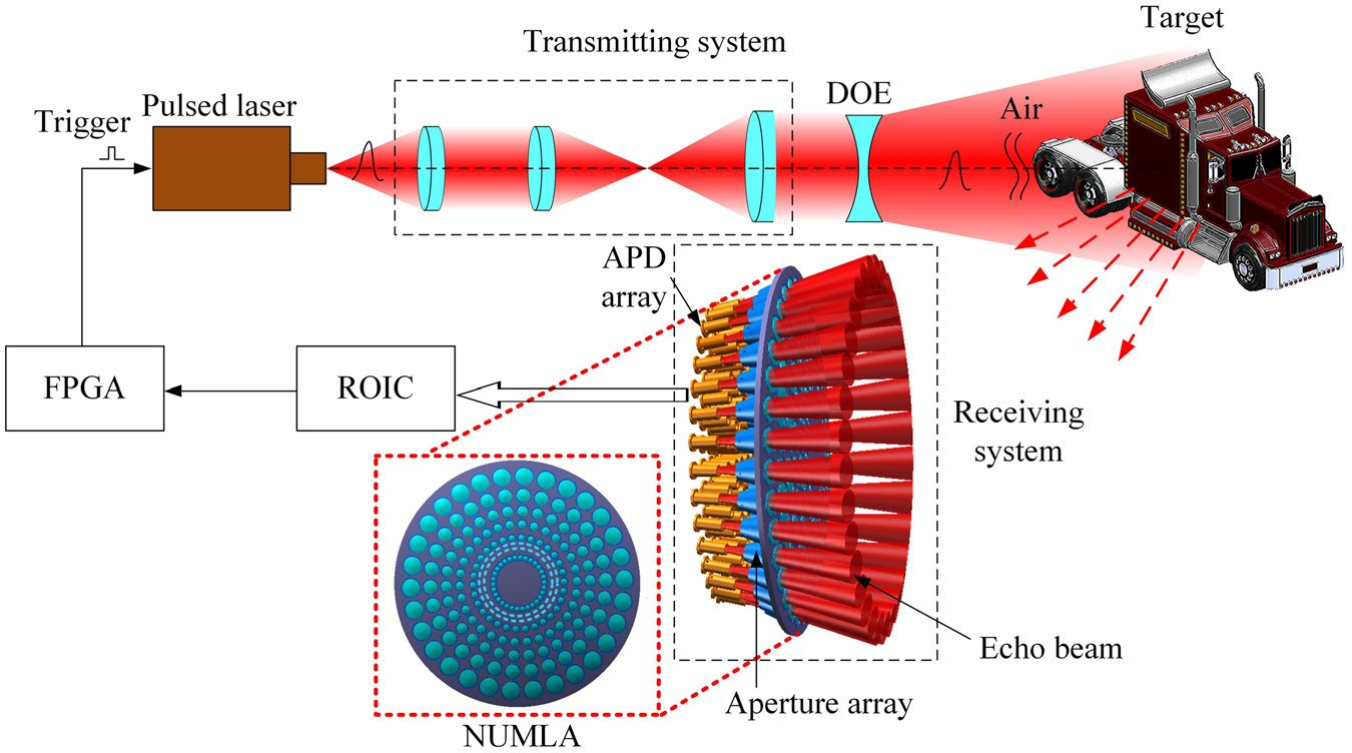
Principle of the proposed system. DOE-diffractive optical element, ROIC-readout integrated circuit, APD-avalanche photodiode, NUMLA-non-uniform microlens array. A DOE is used to enlarge the FOV of transmitting system. The reflected or scattered pulsed laser echo beam is focused on the APD array by NUMLA.

Figure 1 depicts the principle of the proposed 3D pulsed laser imaging system inspired by the compound and human hybrid eye. The system includes a field programmable gate array (FPGA), a pulse laser, a transmitting system, a target, a receiving system, and a readout integrated circuit (ROIC). The working flow is described as follows.

(1) The FPGA emits an electronic pulse to trigger the pulsed laser, and then the pulsed laser launches a pulsed laser beam under the command of the electronic pulse. This moment is regarded as the start timing moment for a timer in the FPGA.

(2) The pulsed laser beam is collimated and expanded by a transmitting system. A diffractive optical element (DOE) is used in front of the transmitting system to enlarge the FOV of the transmitting system and homogenize the intensity of the transmitting pulsed laser beam. Then, the transmitting pulsed laser beam projects and illuminates to a target surface.

(3) The transmitting pulsed laser beam is reflected or scattered by the target surface and the reflected or scattered pulsed laser echo beam is received by a receiving system. The receiving system consists of a non-uniform microlens array (NUMLA), an APD array, and an aperture array. The NUMLA consists of *N* ring microlenses and there are *M* single microlenses present in each ring. These microlenses (*N*×*M*) segment the echo beam into the different optical channels and are detected by the corresponding APD array. An aperture array ensures that one microlens corresponds to one APD detector and prevents optical crosstalk between adjacent optical channels.

(4) The APD array responses the echo beam and outputs the electronic signal. The electronic signals are collected and processed by the ROIC. The ROIC can obtain the stop timing moments of the processed electronic signals, and these moments are recorded by the timer in the FPGA. The time of flight between the target and the pulsed laser is obtained through subtraction of the stop timing moments and the start timing moment. Hence, a 3D image of the target is obtained by the proposed system.

### System modeling

**Figure 2:**
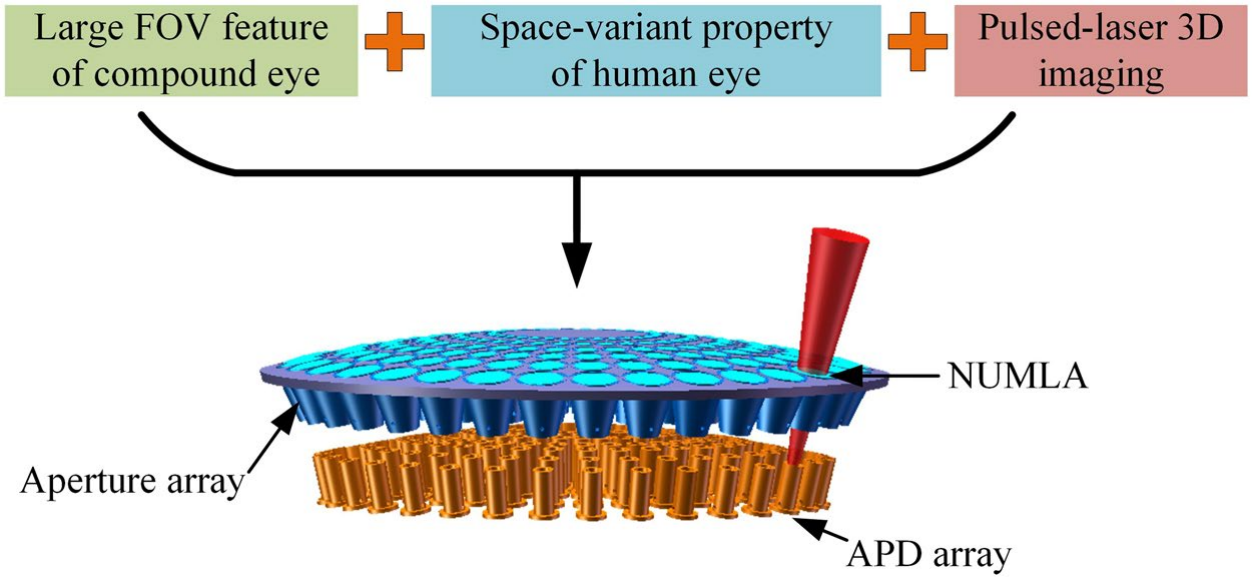
Receiving system that possesses large FOV and space-variant resolution simultaneously. Compound and human eyes offer large FOV feature and space-variant resolution property, respectively.

As mentioned in the principle section, the receiving system is a core component of the proposed imaging system. Therefore, we present the detailed modeling of the receiving system. The receiving system combining the large FOV feature of the compound eye and the space-variant resolution property of the human eye simultaneously is shown in Fig. 2. The microlenses of the NUMLA are distributed on a curved surface to mimic the large FOV feature of the compound eye. Each microlens of the NUMLA is an optical channel mimicking the ommatidia of the compound eye. Each optical channel only transfers a portion of the FOV, and the large FOV is achieved by adding a portion of the FOV of these microlenses.

**Figure 3:**
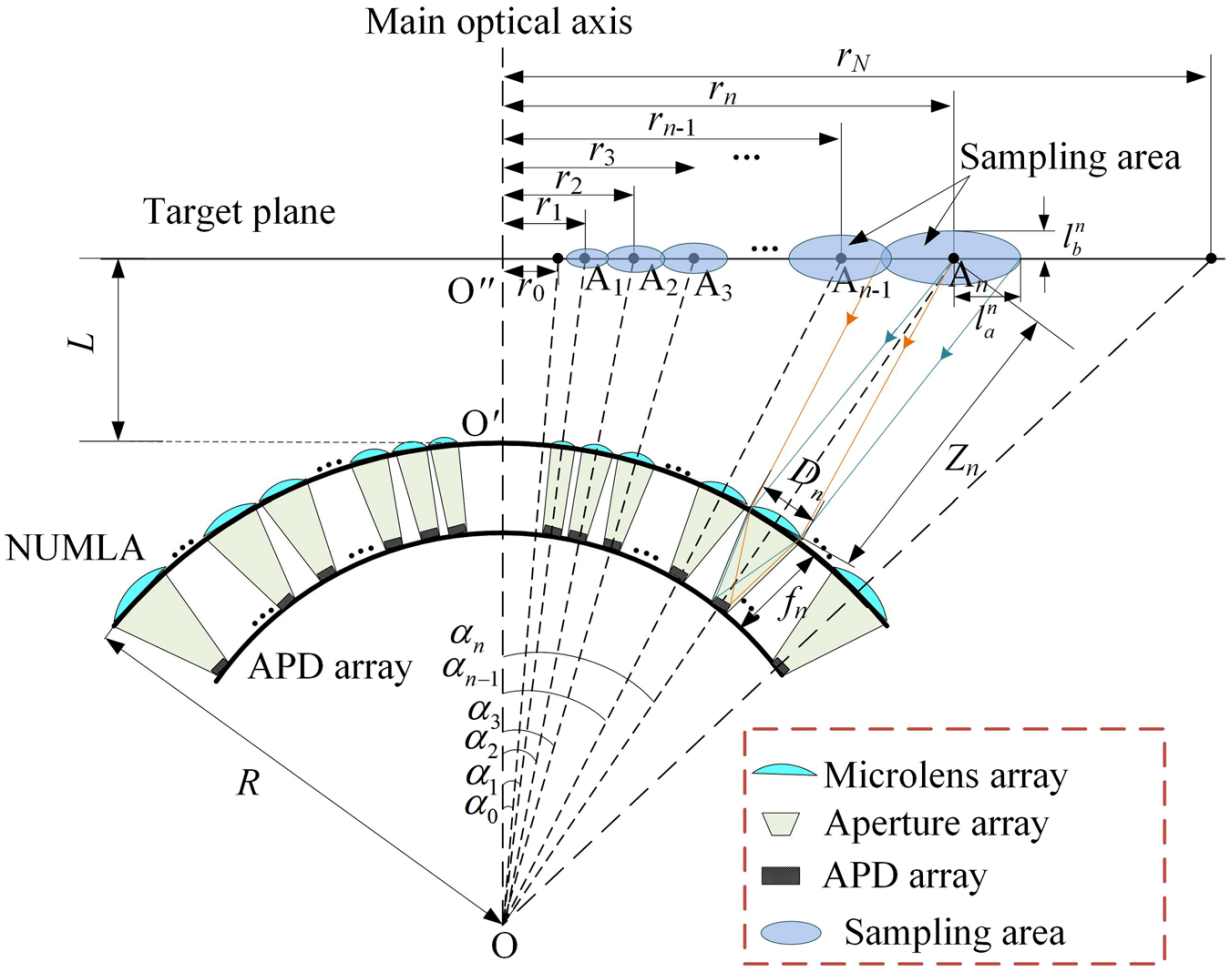
Geometrical model of the receiving system. We suppose that the radius of the curved surface is *R*, and the distance between the target plane and the NUMLA is *L*. *α*_0_ is the angle between the blind area and the main optical axis, and *α*_*n*_ is the angle between the main optical axis and the *n*-th ring microlens of the NUMLA.

Figure 3 shows the geometrical model of the receiving system. Different from our previous model^30^, the space-variant resolution, i.e., the retina-like property of the human eye in the proposed method is that the distance between the main optical axis and the central point distribution of the sampling area for these microlenses meets a geometric sequence growth.

We assume that the blind radius of the target plane is *r*_0_, and the distance between the main optical axis and the central point of sampling area for the *n*-th ring microlens is *r*_*n*_. The position of the *n*-th ring microlens on the curved surface is described as^43^.

1 $$\{\begin{array}{lll}{r}_{1}=\frac{{r}_{0}}{1-\,\sin \,(\pi /M)}, & \,{r}_{n}={r}_{1}{q}^{n-1}, & q=\frac{1+\,\sin (\pi /M)}{1-\,\sin \,(\pi /M)}\\ \tan \,{\alpha }_{n}=\frac{{r}_{n}}{R+L}, & (1\le n\le N) & \end{array}$$

Meanwhile, the optical parameters of these microlenses on the curved surface are non-uniform. For the *n*-th ring microlens, the optical parameters can be calculated by

2 $$\{\begin{array}{c}\frac{1}{{z}_{n}}+\frac{1}{v}=\frac{1}{{f}_{n}}=({n}_{r}-1)(\frac{1}{{g}_{n}}-\frac{1}{R})\,\,\,\\ {D}_{n}=\frac{2{r}_{0}\,\sin \,(\pi /M){[1+\sin (\pi /M)]}^{n-1}}{{[1-\sin (\pi /M)]}^{n}}-\frac{{\rm{\Delta }}d[(L+R)/\cos ({\alpha }_{n})-R]}{{f}_{n}}\\ {h}_{n}={g}_{n}-R[1-\,\cos ({\beta }_{n})]-\sqrt{{{g}_{n}}^{2}-{R}^{2}\,{\sin }^{2}({\beta }_{n})},\,{\rm{where}}\,\sin ({\beta }_{n})=\frac{{D}_{n}}{2R}\end{array},$$

where *D*_*n*_, *f*_*n*_, *h*_*n*_, *g*_*n*_, *n*_*r*_, Δ*d*, and *v* denote the aperture, focal length, thickness, curvature radius, refractive index of the *n*-th ring microlens, size of the APD detector and image distance, respectively. Because the microlens is placed on the curved surface, the sample area of a single microlens on the target plane is an ellipse^30^. For the *n*-th ring microlens, the semi-major *l*_*n*_^*a*^ and semi-minor axis *l*_*n*_^*b*^ of the ellipse can be obtained by

3 $$\{\begin{array}{c}{l}_{a}^{n}=\frac{[\frac{L+R}{\cos ({\alpha }_{n})}-R]\tan (\frac{{\rm{\Delta }}d}{2{f}_{n}})+\frac{{D}_{n}}{2}}{\cos ({\alpha }_{n})}\\ {l}_{b}^{n}=\frac{{r}_{0}\,\sin \,(\pi /M){[1+\sin (\pi /M)]}^{n-1}}{{[1-\sin (\pi /M)]}^{n}}\end{array}.$$

From the analysis above, the entire geometrical model of the receiving system can be established. For pulsed-laser 3D imaging, the transmitting laser pulse beam is a function of Gaussian shape in terms of time-domain, which is written as

4 $${P}_{t}(t)=\frac{{E}_{t}}{\tau \sqrt{2\pi }}\,\exp (\frac{-\,{t}^{2}}{2{\tau }^{2}}),$$

where *E*_*t*_ is the original pulse energy, and *τ* is the transmitting pulse width. The transmitting pulsed laser beam projects to the target. For an area target with uniform illumination and reflectivity, the power scattered by the target in the backward direction is given by^3^

5 $${P}_{sc}={\rho }_{\pi }{I}_{t}{A}_{t},$$

where *ρ*_π_ is the target reflectivity per steradian in the backward direction, *I*_*t*_ is the intensity of the transmitted pulse beam at the target location, and *A*_*t*_ is the area of the target. Under the assumptions that the target is normal and its scattering is Lambertion, i.e., *ρ*_π_ = *ρ*_*t*_/π, where *ρ*_*t*_ is the total hemispherical reflectivity. The transmitter intensity is flat over the entire illuminated region at the target plane, i.e., *I*_*t*_ = *P*_*tar*_/*A*_illum_. *P*_*Tar*_ is the optical power at the target location, and *A*_illum_ is the area illuminated. They can be obtained by

6 $$\{\begin{array}{c}{P}_{tar}={\eta }_{tran}\times {\eta }_{atm}\times {\eta }_{DOE}\times {P}_{t}\\ {A}_{illum}=\pi {w}_{L}^{2}=\pi \{{[L\times \tan (\theta /2)]}^{2}+\langle {W}_{turb}^{2}\rangle \}\end{array},$$

where *η*_*trans*_, *η*_*atm*_, *η*_*DOE*_, *w*_*L*_, and *θ* are the transmitting system efficiency, one-way atmospheric transmission, over efficiency of the DOE, beam radius at the target plane, and full diffusion angle of the DOE, respectively. 〈*W*_*turb*_〉 is the increased beam radius caused by atmospheric turbulence, which can be expressed as^44^

7 $$\langle {W}_{turb}^{2}\rangle =2.18\,{C}_{n}^{2}{l}_{0}^{-1/3}{L}^{3}$$

where 〈 〉 denotes the ensemble average, *l*_0_ is the inner-scale size of turbulence, and *C*_*n*_^2^ is the index of refraction structure constant. The scale of *C*_*n*_^2^ represents the intensity of the atmospheric turbulence.

The one-way atmospheric transmission *η*_*atm*_ affected by the atmospheric condition can be evaluated by Beer-Lamber law, which is written as

8 $${\eta }_{atm}=\frac{I(\lambda ,L)}{I(\lambda ,0)}=\exp [-\,{\delta }_{T}(\lambda )L],$$

where *I*(λ, *L*) is optical intensity at target plane, *I*(λ, 0) is optical intensity at transmitter location, and *δ*_*T*_(λ) is extinction coefficient. The extinction coefficient has the following relationship with the laser wavelength and visibility^45^.

9 $${\delta }_{T}(\lambda )=\frac{3.91}{{R}_{v}}{(\frac{\lambda }{550})}^{-q},$$

where *Rv* is the meteorological visibility in km^−1^, λ is the optical beam wavelength in nanometers, and *q* is the correction factor. The correction factor depends on the meteorological visibility and can be written as

10 $$\{\begin{array}{lll}q=1.6 &  & {\rm{when}}\,{R}_{v} > 50\,{\rm{km}}\\ q=1.3 &  & {\rm{when}}\,6\,{\rm{km}} < {R}_{v} < 50\,{\rm{km}}\\ q=0.585\,{{R}_{v}}^{1/3} &  & {\rm{when}}\,{R}_{v} < 6\,{\rm{km}}\end{array}.$$

The total optical power received by the *n*-th ring APD detector is given by

11 $${P}_{r}^{n}={P}_{sc}\times \frac{{A}_{rec}}{{{z}_{n}}^{2}}\times {\eta }_{atm}\times {\eta }_{rec},$$

By substituting Eqs (5) and (6) into Eq. (11), we can obtain the pulsed echo profile for the *n*-th ring APD detector as

12 $$\begin{array}{rcl}{P}_{r}^{n} & = & \frac{{\rho }_{t}}{\pi }\times \frac{{P}_{tar}}{{A}_{illum}}\times {A}_{t}\times \frac{{A}_{rec}}{{z}^{2}}\times {\eta }_{atm}\times {\eta }_{rec}\\  & = & {P}_{t}(t-2\frac{L}{c})\times \frac{{\rho }_{t}\times {A}_{t}\times {A}_{rec}}{{A}_{illum}\times \pi {{z}_{n}}^{2}}\times {\eta }_{atm}^{2}\times {\eta }_{DOE}\times {\eta }_{rec}\times {\eta }_{tran}.\\  & = & \frac{{E}_{t}}{{\tau }_{t}\sqrt{2\pi }}\exp [-\,\frac{1}{2{{\tau }_{t}}^{2}}{(t-2\frac{L}{c})}^{2}]\times \frac{{\rho }_{t}\times {A}_{t}\times {A}_{rec}}{{A}_{illum}\times \pi {{z}_{n}}^{2}}\times {\eta }_{atm}^{2}\times {\eta }_{DOE}\times {\eta }_{rec}\times {\eta }_{tran}\end{array}$$

In Eq. (12), λ_*t*_ is the pulse width after broadening by the atmospheric turbulence, which is expressed as^44^

13 $$\{\begin{array}{rcl}\langle {\tau }_{t}^{2}\rangle  & = & {{\tau }_{0}}^{2}+\frac{8\times (0.3908\,{C}_{n}^{2}\,L{L}_{0}^{5/3})}{{c}^{2}}\\ {L}_{0} & = & 5/1+{(\frac{L-7500}{2000})}^{2}\end{array}$$

where *L*_0_ is the outer-scale size of turbulence.

### SNR analysis

SNR is an important parameter of pulsed-laser 3D imaging because it affects the detection range and accuracy. The SNR equation of the proposed system for the *n*-th ring APD detector can be expressed by^46^

14 $$SNR=\frac{{P}_{sig}^{n}}{{P}_{th}+{P}_{a}+{P}_{dark}+{P}_{shot}+{P}_{back}},$$

where *P*^*n*^_*sig*_ is the signal power output by the *n*-th ring APD detector, *P*_*th*_ is the mean-squared thermal-noise power, *P*_*a*_ is the mean-squared noise power added by the electronic amplifier, *P*_*dark*_ is the mean-squared dark-current-noise power generated by the leakage current, *P*_*shot*_ is the mean-squared signal shot noise power, and *P*_*back*_ is the output power of background noise produced by solar illumination.

The signal power output by the APD detector is given by

15 $${P}_{sig}^{n}={(M{P}_{r}^{n}{\rho }_{D})}^{2}{R}_{L},$$

where *M* is the current gain of the APD detector*, ρ*_*D*_ = *η*_*D*_*e*/*hf* is the current responsivity of the APD detector, *η*_*D*_ *is* the quantum efficiency of the APD detector, *e* is the electron charge, *h* is Planck’s constant, *f* = *c/λ* is the frequency of light, and *R*_*L*_ is the effective load resistance of the APD detector.

The terms of the noises in Eq. (14) are described as

16 $$\{\begin{array}{rcl}{P}_{th} & = & 4kTB\\ {P}_{a} & = & 4k{T}_{a}B\\ {P}_{dark} & = & 2e{I}_{dark}{M}^{2}{F}_{ex}B{R}_{L}\\ {P}_{shot} & = & 2e{P}_{r}^{n}{M}^{2}{F}_{ex}{\rho }_{D}B{R}_{L}\\ {P}_{back} & = & 2e{P}_{BK}{M}^{2}{F}_{ex}{\rho }_{D}B{R}_{L}\\ {P}_{BK} & = & S{}_{irr}\,{\rm{\Delta }}\lambda {{\rm{\Omega }}}_{R}{\rho }_{t}{\eta }_{rec}{A}_{rec}\end{array},$$

where *k* is Boltzmann’s constant, *B* is the electrical bandwidth of the system, *T* is the temperature in Kelvin, *T*_*a*_ is the effective noise temperature, *I*_*dark*_ is the dark current, *F*_*ex*_ is the excess-noise factor, *S*_*irr*_ is the solar irradiance, Δλ is the optical bandwidth of the receiver, and *Ω*_*R*_ is the receiver FOV of the APD detector.

By substituting Eqs (15) and (16) into Eq.(14), we can obtain the expression of the SNR of the APD detector, which can be written as

17 $$SN{R}_{n}=\frac{{P}_{r}^{n2}{\rho }_{D}^{2}{M}^{2}}{\frac{4kB(T+{T}_{a})}{{R}_{L}}+2e{M}^{2}{F}_{ex}B({P}_{r}^{n}{\rho }_{D}+{P}_{BK}{\rho }_{D}+{I}_{dark})}.$$

## Results

We carry out simulation experiments to verify the effectiveness of the proposed imaging system. The experiments include imaging with large FOV to mimic the compound eye and imaging with space-variant resolution, rotation and the scale invariances to imitate the retina-like property of the human eye. We also analyze the SNR of the proposed imaging system.

### Simulation parameters

Table 1 shows the relevant parameters of the simulation experiments. In accordance with these parameters, the entire geometrical structure parameters of the receiving system can be obtained using Eqs (1 and 2), including the location distributions of the microlenses on the curved surface (the angle between the main optical axis and the microlens) and the optical parameters (the focal length, curvature radius, aperture, and thickness) of the microlenses of the NUMLA. The beam-expanding ratio of the transmitting system is 3×. The full diffusion angle of the DOE is about 62º. The pulse energy of the pulsed laser should be larger than that calculated by Eq. (11) when the power of the pulsed echo profile equals the minimum detectable input power of the APD detector. A 1550 nm wavelength of the pulsed laser is chosen because of its eye-safe wavelength. The simulated distance (1 m) is less than that of traditional pulsed-laser 3D imaging system because that the FOV of the proposed system is up to 52°. Under this FOV, it is a challenge to find a target with appropriate sizes to be imaged at a long distance. For example, when the distance is 100 m and the FOV is 52°, the appropriate circumradius of the target is up to about 49 m (100 m×tan(52°/2)). Therefore, we select this distance (1 m) as the simulated distance. Users can adjust the FOV according to the desired distance and the size of the imaged target. The numbers of rings and sections of the microlens are set to 18 and 30 for achieving a large optical factor.

**Table 1:** Parameter values used in the simulations.

Parameter type	Abbreviation	Value	Parameter type	Abbreviation	Value
Pulse energy	*E*_*t*_	20 μJ	Refractive index	*n*_*r*_	1.517
Wavelength	*λ*	1550 nm	Size of the APD detector	Δ*d*	100 μm
Initial pulse width	*τ*_0_	1 ns	Image distance	*v*	150 mm
Initial beam radius	*W*_0_	0.02 m	Blind radius	*r*_0_	20 mm
Distance	*L*	1 m	Rings	*N*	18
Radius of the curved surface	*R*	0.6 m	Sectors	*M*	30

**Figure 4:**
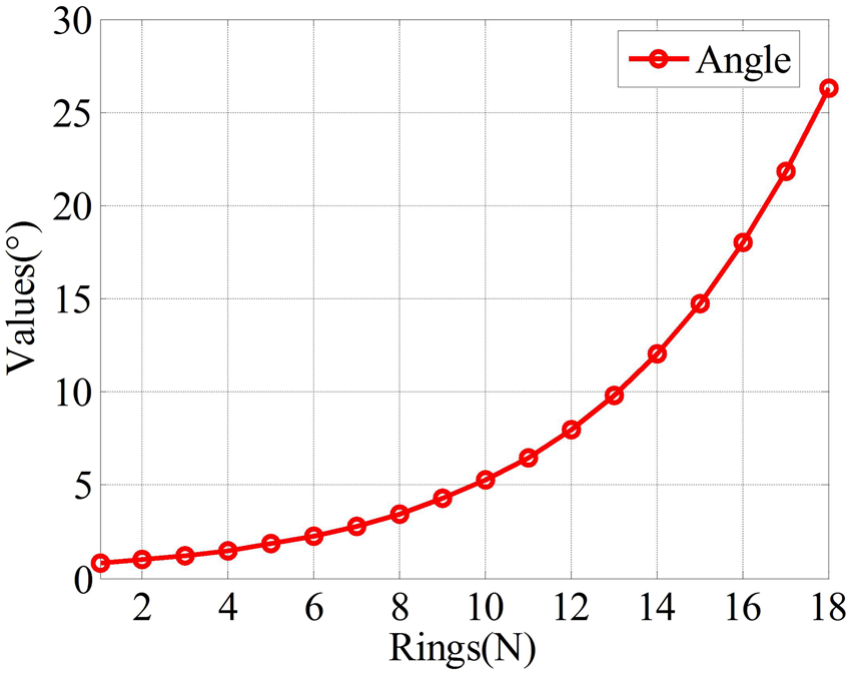
Angle between the main optical axis and the microlens.

**Figure 5:**
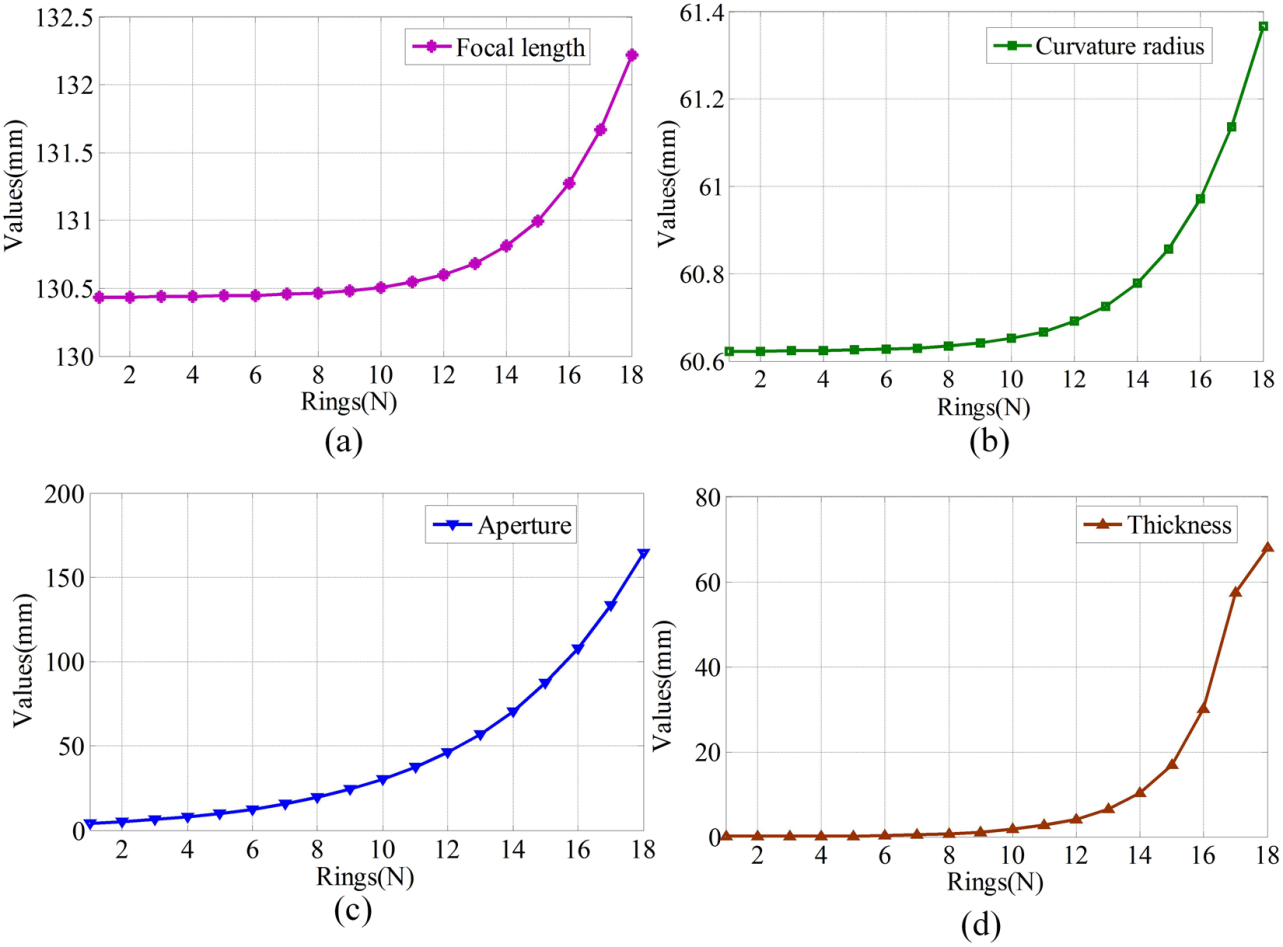
Optical parameters of the microlenses of the NUMLA.

Figures 4 and 5 show the results of the angle between the main optical axis and the microlens and the optical parameters of these microlenses, respectively. From Fig. 4, we find that the angle increases from 0.8° to 26° when the number of rings increases from 1 to 18. Therefore, the entire FOV of the proposed imaging system is 52°(2 × 26°). The figure also shows that the location distributions of the microlenses on the curved surface are non-uniform. The distribution of the microlens near the main optical axis is more compact than that of the microlens far from the main optical axis.

As shown in Fig. 5, we find that: (1) The focal length increases from 130.4 mm to 132.2 mm when the number of rings increases from 1 to 18. (2) The curvature radius increases from 60.6 mm to 61.4 mm when the number of rings increases from 1 to 18. (3) The aperture increases from 4 mm to 164 mm when the number of rings increases from 1 to 18. It indicates that the FOV for the microlens that far from the main optical axis is larger than that near the main optical axis. (4) The thickness increases from 0.1 mm to 68 mm when the number of rings increases from 1 to 18.

### Imaging with a large FOV and space-variant resolution

**Figure 6:**
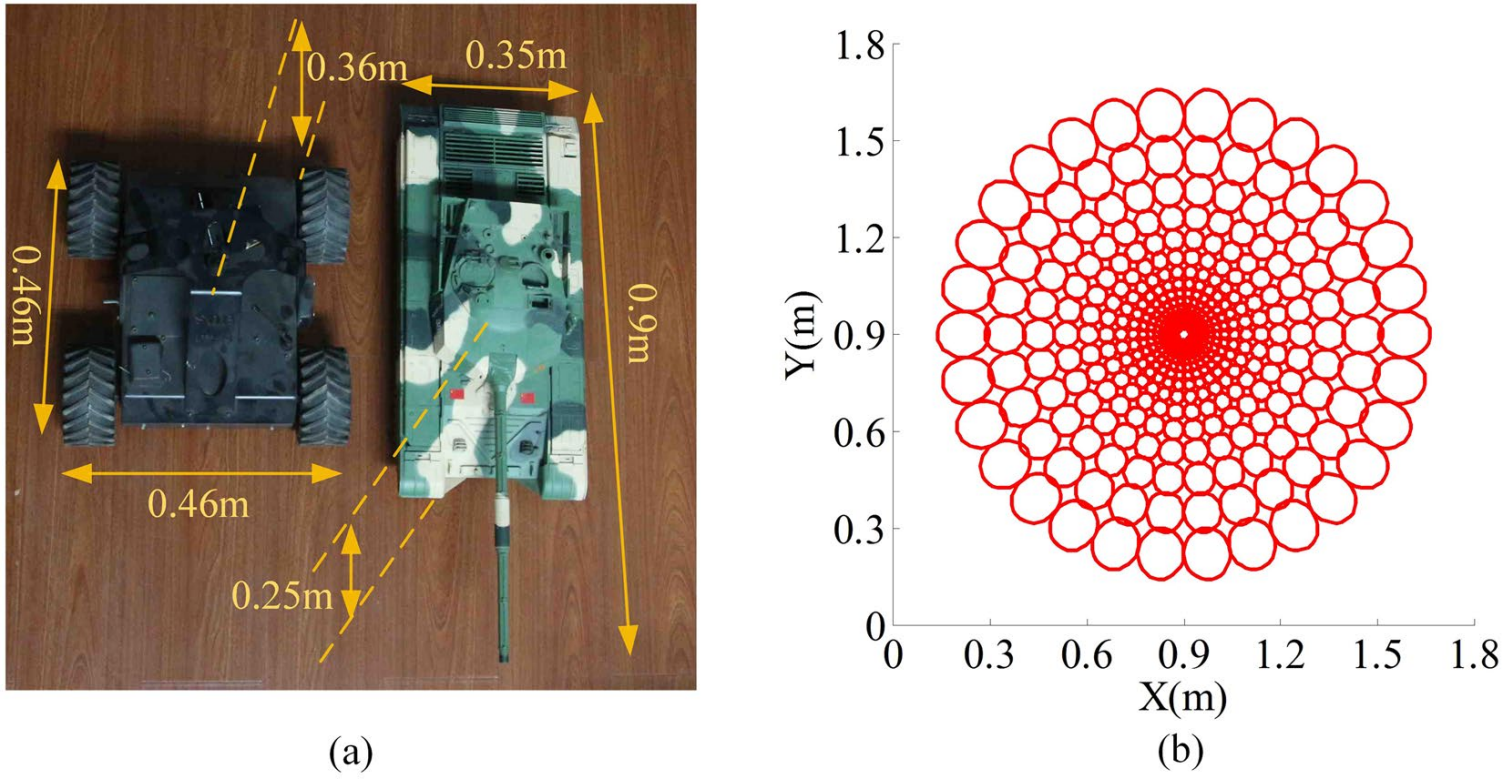
(**a**) Two targets used in the simulation experiment. The tank model is the interested target and the car model is the uninterested target. (**b**) Sampling areas on the target plane.

We carry out a simulation experiment to verify that the proposed system possesses the large FOV feature of the compound eye, i.e., imaging with large FOV, and has the space-variant resolution property of the human eye. Two targets (a tank model and a car model) are used in the simulation experiment. The sizes of the tank and the car models are 0.9 m × 0.36 m × 0.25 m (length × width × height) and 0.46 m × 0.46 m × 0.36 m (length × width × height) respectively, as shown in Fig. 6(a). The reflectivities of the tank and the car models are approximately 40%, and 10%, respectively. The FOV of the proposed system is 52°, thus, the beam diameter is approximately 1 m when the distance between the targets and the transmitting system is 1 m. Therefore, the beam diameter is capable to cover the area of these two targets for imaging. The tank model is regarded as the interested target, and the car model is the uninterested target.

From the principle of the proposed system and the parameters in Table 1, the sampling areas of each microlens on the target plane can be obtained and are shown in Fig. 6(b). From Fig. 6(b), we find that these sampling areas are ellipses and the resolution is the highest in the central area as well as decreases monotonically toward the periphery area. We can also find that there are some overlaps and blind areas between adjacent sampling areas. The optical fill factor is up to 91% by substituting Eq. (3) into our previous paper^30^.

**Figure 7:**
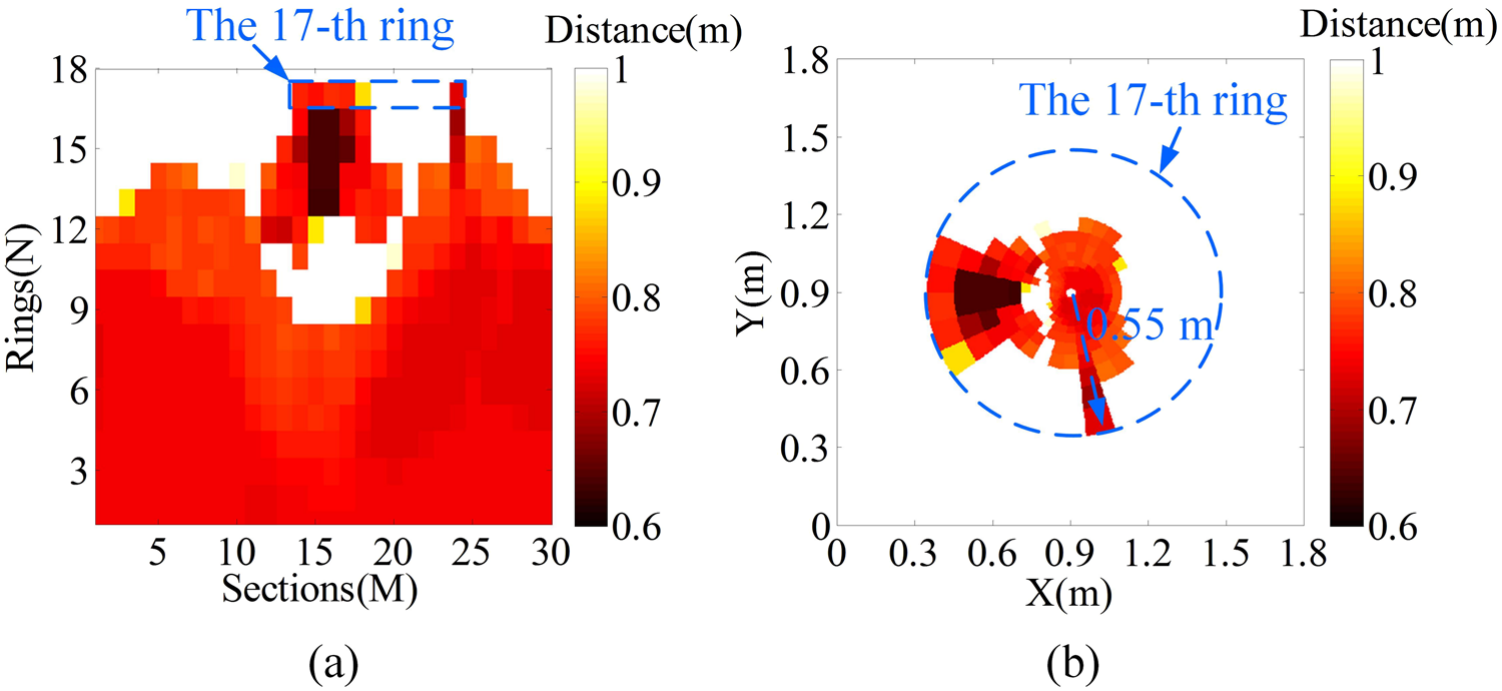
(**a**) LPT image. (**b**) Reversed LPT image.

The APD array receives the echo beam in the sampling area and generates the electronic pulse signal. We use typical peak discriminator to obtain distance information by the time of flight for simplifying the simulation experiments. The number of the APD array is the same as that of the microlenses. Hence, the APD array also consists of 18 rings and 32 sections in each ring. Each detector of the APD array achieves the distance information of the corresponding ellipse sampling area of the targets. The axes of traditional LPT image (eccentricity and angle) can be represented by rings and sections respectively in this paper because *r*_*n*_ already meets the geometric sequence growth according to Eq. (1). Therefore, the LPT image of the distance information is obtained by circularly readout, i.e., the sections and rings are referred to as the horizontal and vertical axes respectively. Figure 7(a) shows the LPT image of the two targets. Through the transformation from the LPT to Cartesian coordinate, the reversed LPT image can be achieved and is shown in Fig. 7(b). Figure 7(b) shows that the circumcircle radius of the tank model target imaged by the proposed system is approximately 0.55 m. Therefore, the entire FOV of the proposed system is more than 38° (2 × arctan [0.55/(*R* + *L*))]), which indicates that the proposed system can realize imaging with a large FOV mimicking the compound eye. The simulated entire FOV of the proposed system (38°) is narrower than that of the theoretical designed (52°) because the tank model is fully covered by the 17 ring microlenses and no target is in the FOV of the outermost ring microlenses, as shown in Fig. 5(a). The center of the interested tank model has a high resolution and the uninterested car model has a low resolution. Therefore, the high resolution benefits the recognition of the tank model, whereas the low resolution of the uninterested car model provides context and situation awareness. The results indicate that the proposed system can realize imaging with space-variant resolution mimicking the retina-like property of the human eye.

### Rotation and scaling invariance

**Figure 8:**
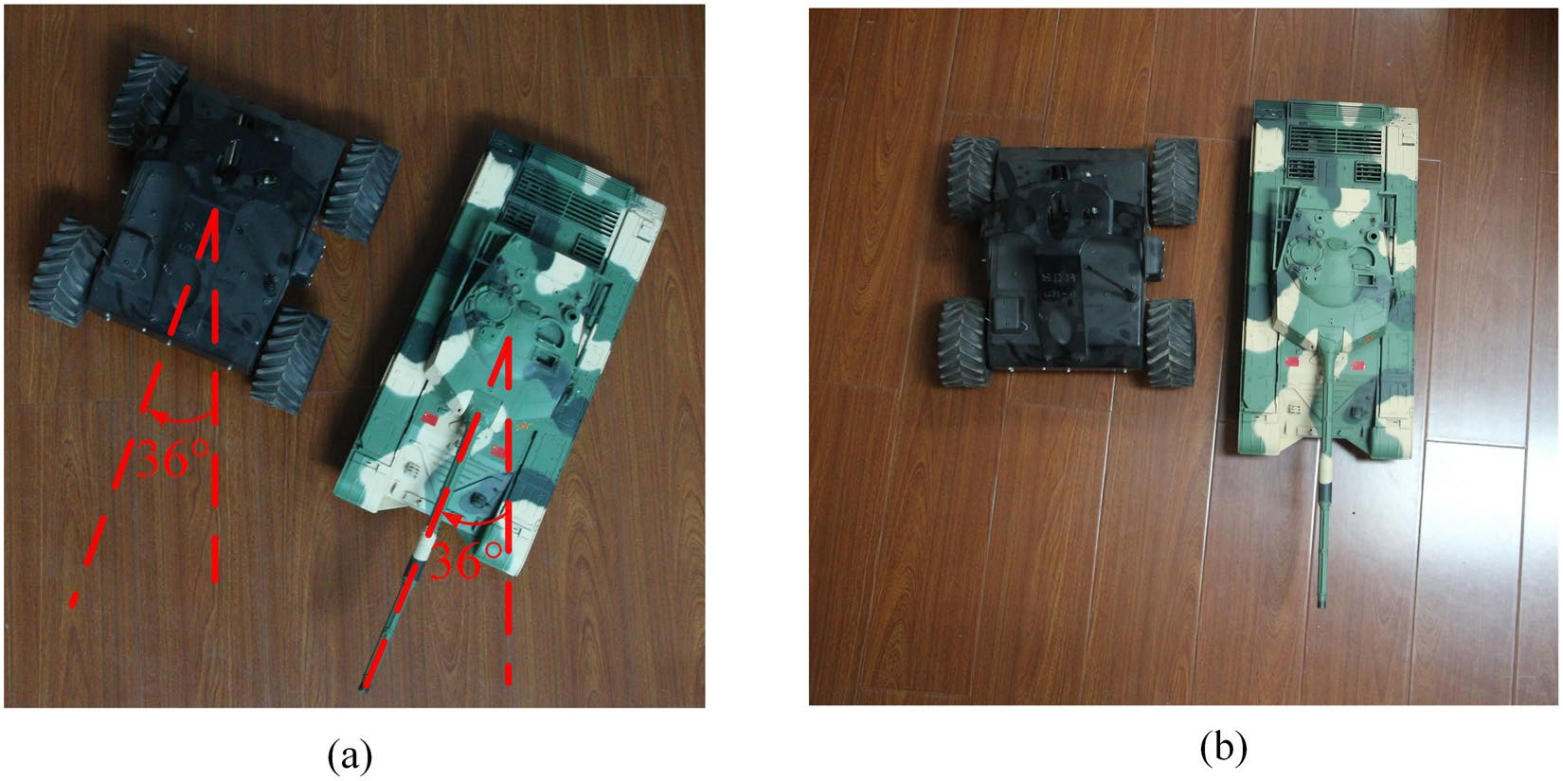
(**a**) Two targets clockwise rotated by 36°. (**b**) Two targets decreased to 0.7 times the original size.

**Figure 9:**
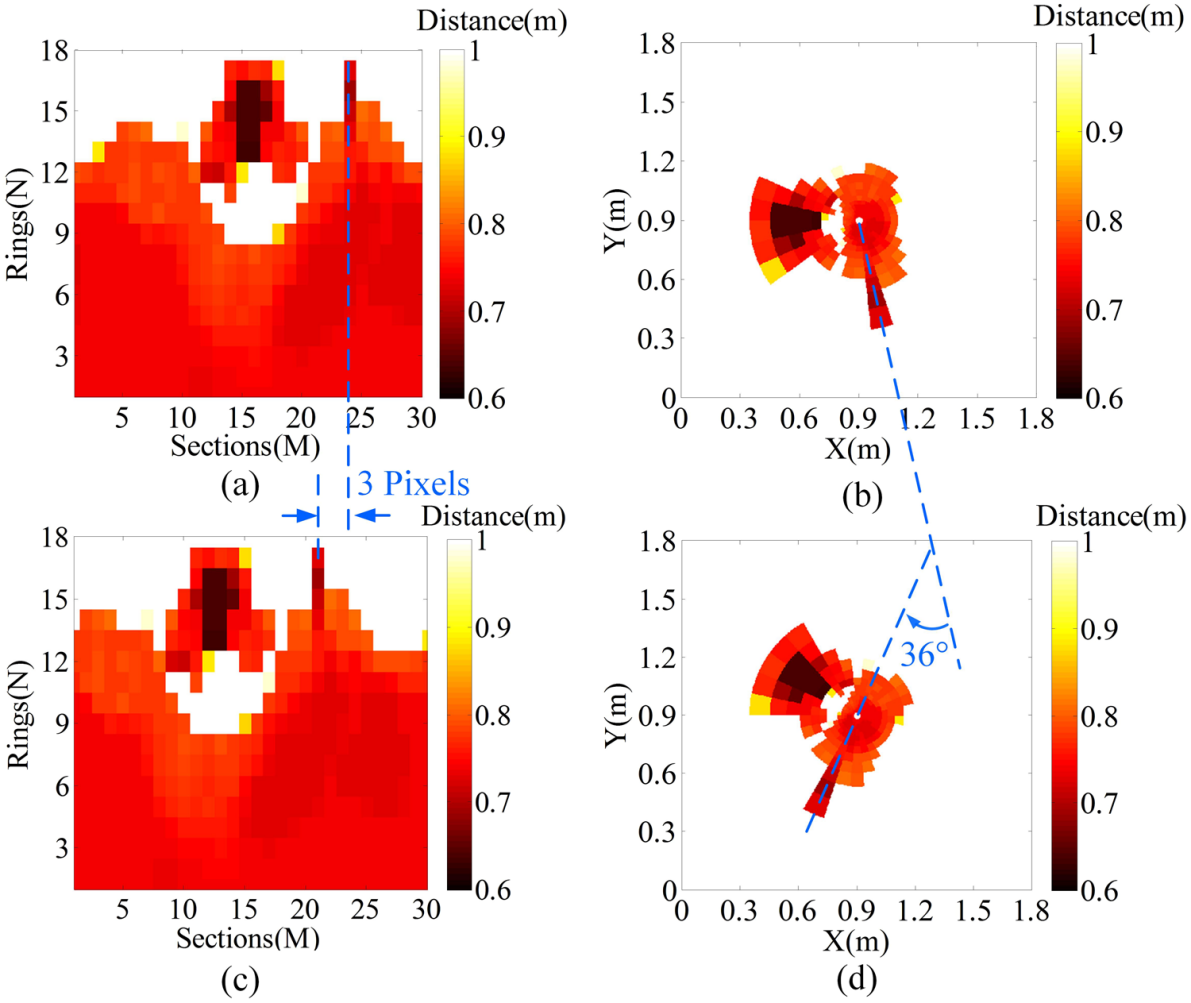
Results of the rotation invariance. (**a**) and (**b**) are the LPT and reversed LPT images before rotating by 36°, respectively. (**c**) and (**d**) are the LPT and reversed LPT images after rotating by 36°, respectively.

**Figure 10:**
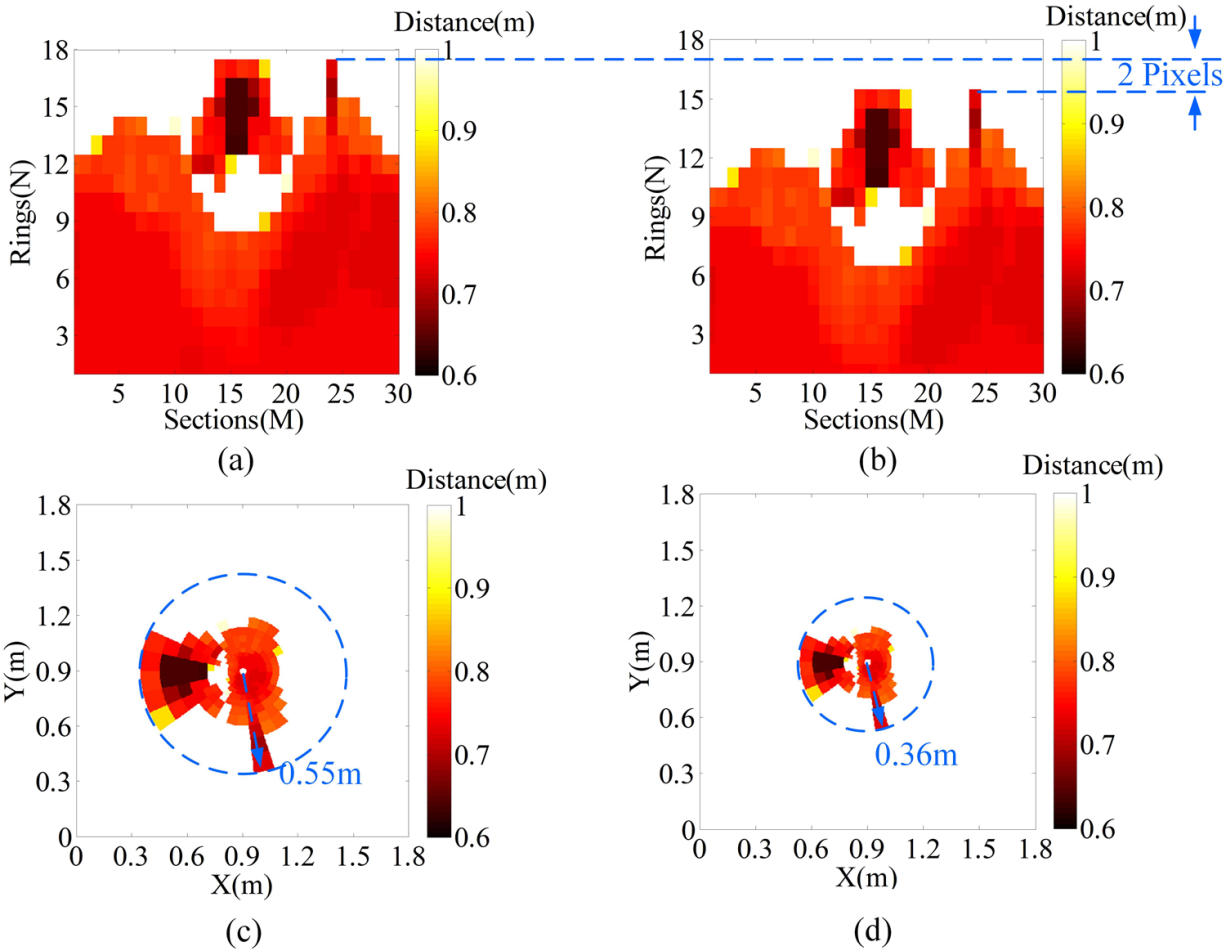
Results of scaling invariance. (**a**) and (**b**) are the LPT images before and after scaling, respectively. (**c**) and (**d**) are the reversed LPT images before and after scaling, respectively.

We carry out the verification experiments on the proposed system to test the rotation and scaling invariance properties under the aforementioned simulation conditions in this section. The two targets (tank and car models) are clockwise rotated by 36°, as shown in Fig. 8(a) to test the rotation invariance. We also decrease the sizes of the tank and the car models to 0.7 times their original size to test the scaling invariance, as shown in Fig. 8(b). Figure 9 shows the LPT and reversed LPT images before and after rotating 36°, and Fig. 10 shows the LPT and reversed LPT images before and after scaling.

Comparison between Fig. 9(a,c) shows that the LPT image after rotating the targets by 36° has not changed significantly. Only a shift of 3 pixels is observed along the sections axis. The shift pixel is theoretically obtained as 3 (36°/360° × *M*) using simple calculation. The results show that the simulation result is in good agreement with the theoretical result. Figure 9(b,d) show the difference between the reversed LPT image before and after rotating is that the targets are rotated by 36°, which indicates the proposed system has the rotation invariance of the human eye.

Comparison between Fig. 10(a,b) shows that the LPT image after scaling the targets to 0.7 times their original size also has not changed. Only a shift of 2 pixels is found along the sections axis. The shift pixel along the sections axis is theoretically obtained as 1.72, which is equal to −1 × log (0.7)/log (*q*), where *q* is the increase coefficient in Eq. (1) and equals 1.23. The radius of the circumcircle covering the two targets before scaling is 0.55 m as shown in Fig. 10(c) and that after scaling is 0.36 m as shown in Fig. 10(d). The scaling ratio is 0.65, and the relative error is approximately 7% [(0.7−0.65)/0.7]. The results show that the simulation result is in good agreement with the theoretical result, which indicates that the proposed system has the scaling invariance of the human eye.

### SNR

**Figure 11:**
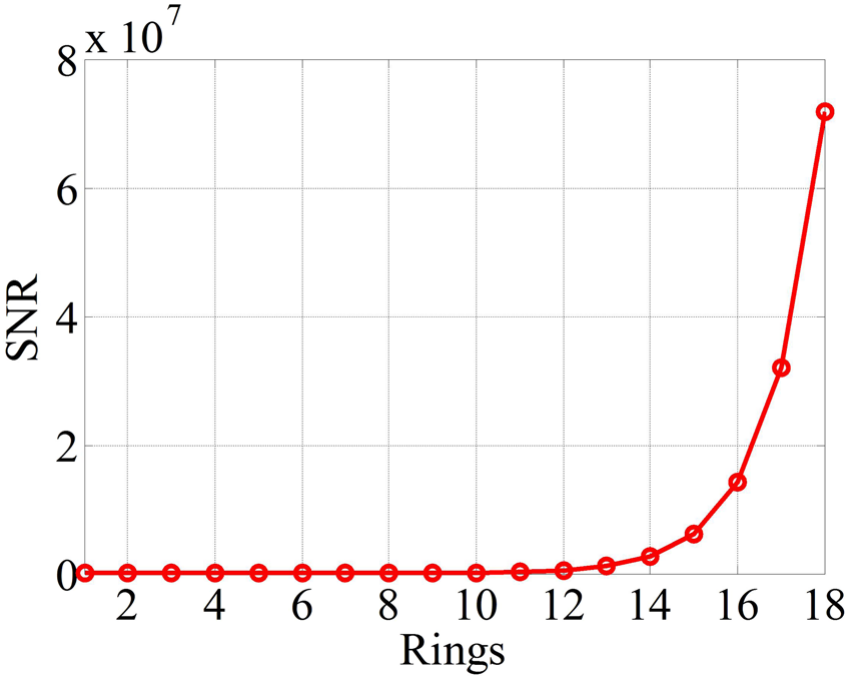
SNR of the proposed system in each ring.

To achieve the SNR of the proposed system, typical parameters of the APD detector in Eq.(14) are set as follows: *η*_*D*_ = 0.9, *R*_*L*_ = 50 Ω, *B* = 1/(2*τ*), *T* = 300 K, *T*_*a*_ = 175 K, *I*_*dark*_ = 100 nA, *F*_*ex*_ = 10, *S*_*irr*_ = 266 W/m^2^/μm, Δλ = 4 nm, *e* = 1.602 × 10^−19^ C, *h* = 6.63 × 10^−34^ J·s, and *k* = 1.38 × 10^−23^ J/K^46^. Figure 11 shows the results of the SNR of the proposed system in each ring. As shown in the figure, the SNR increases from 3.6 to 7.2 × 10^7^ when the number of rings increases from 1 to 18. The SNR increases as the aperture of the microlenses increases at the outer rings, i.e., the maximum SNR is at the outmost periphery area. Therefore, the laser beam profile should be adjusted to produce the best SNR at the center area. The ratio of the SNR of the pulsed echo profile power between the last ring and the first ring is up to 2 × 10^7^. The reason is mainly that the area of the receiver between the two rings is *A*_rec18_/*A*_rec1_ = [π × (*D*_18_/2)^2^]/[π × (*D*_1_/2)^2^] = 1.77 × 10^3^, and the ratio of the area of the target between the two rings is *A*_*t18*_/*A*_*t1*_ = (π × *l*_*a*_^18^*l*_*b*_^18^)/(π × *l*_*a*_^1^ *l*_*b*_^1^) = 1.4 × 10^3^.

## Discussion

A pulsed-laser 3D imaging system with large FOV inspired by the compound and human hybrid eye is proposed on the basis of the aforementioned results and analysis. From the modeling of the receiving system, we can find that the FOV of the proposed system depends largely on many factors including the radius of the curved surface, the distance between the target plane and the NUMLA, blind radius and the numbers of the rings and sections. The imaging resolution of the proposed system depends on the numbers of the rings and sections. To ensure the ranging accuracy, the bandwidth of the APD detector should be larger than the reciprocal of the pulse width of the pulsed laser. From a practical point of view, the selections of these parameters can be adjusted by the users depending on the actual situation. After these aforementioned parameters are set, the FOV and the resolution of the proposed system are determined. It is worth noting that the proposed system still possesses the FOV feature of the compound eye and the space-variant resolution property of the human eye under variation in the distance between the target and NUMLA. When the distance varies, the FOV is the same as before and the proposed system retains the space-variant resolution property and the increase coefficient between adjacent rings. However, the sampling area of each microlens of the NUMLA on the target plane is different from that before the variation. We suppose that the target plane moves closer to the NUMLA with Δ*L*, and then the blind radius and sampling area can be obtained by the following equation

18 $$\{\begin{array}{rcl}{r^{\prime} }_{0} & = & \frac{{r}_{0}}{R+L}\times (R+L-{\rm{\Delta }}L)\\ {r^{\prime} }_{n} & = & {r^{\prime} }_{0}{q}^{n-1},\,q=\frac{1+\,\sin (\pi /M)}{1-\,\sin (\pi /M)}\,(1\le n\le N)\end{array},$$

where ${r^{\prime} }_{0}$ and ${r^{\prime} }_{n}$ are the blind radius and the distance between the main optical axis and the central point of sampling area for *n*-th ring microlens, respectively.

**Figure 12:**
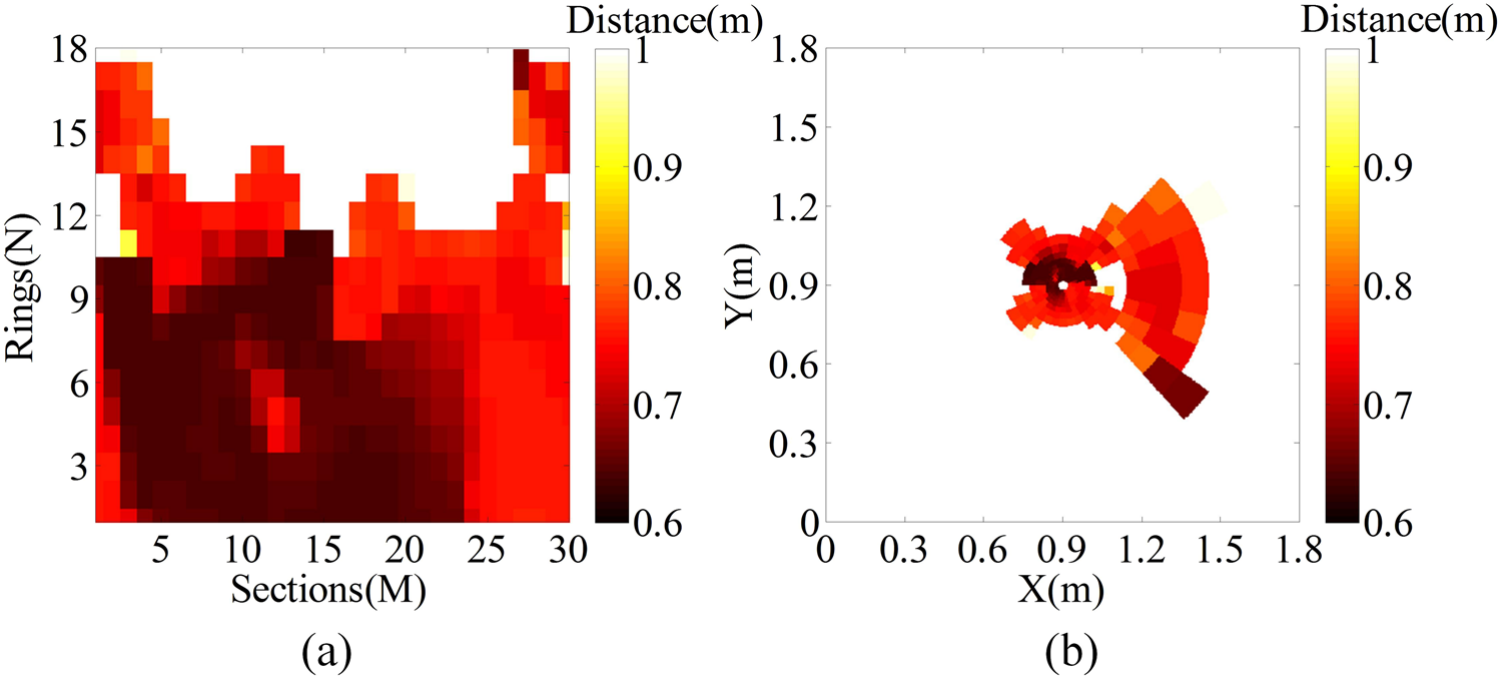
Car model as the interested target and tank model as the uninterested target. (**a**) LPT image. (**b**) Reversed LPT image.

In the aforementioned simulation experiment, the tank model is regarded as the interested target and the car model is the uninterested target. A rotation platform can be used to ensure that the blind area of the receiving system aims at the interested target in a practical situation. For example, when we regard the car model as the interested target and the tank model as the uninterested target, the LPT and reversed LPT images of the two targets are shown in Fig. 12. As shown in this figure, a large number of pixels lie in the interested car model and the car model is easy to be recognized. Fewer pixels represent uninterested the tank mode. Although it is difficult to recognize what it is, it indicates that something exists around the interested car model. If the users want to recognize it, then the blind area of the receiving system should be adjusted to locate it on the target using the rotation platform. Noting that the reversed LPT image has a slight distortion because the overlap areas are twice interpreted. Therefore, we should optimize the structural parameters to reduce the overlap areas.

Unlike the mechanical scanning type Lidar, the proposed system is free of scanning component and uses an APD detector array to detect the pulsed echo signal. Therefore, it can increase the imaging speed significantly. Different from that the traditional flash Lidar using a single lens as the receiver, the proposed method employs microlens array as the receiver. The microlens array is arranged on the curved surface to obtain a large field of view, and the optical parameters of the microlens array are non-uniform to mimic the space-variant resolution property of the human eye. In additional, Compared with the flash Lidar using a single APD integrated array, the proposed system utilizes discrete components to compose the APD array. This system can easily increase the number of the APD detector as required and effectively decrease the electrical crosstalk of adjacent APD detectors due to the existence of the aperture array. The latter task is often difficult for the single APD integrated array. A comparative analysis between this work and some current state-of-art flash Lidar systems is conducted to help understand the characteristics of the proposed system, and the results are listed in Table 2. Notably, the imaging speed mainly depends on the repetition rate of the pulsed laser, the response time of the APD detector, and the processing speed of the ROIC. The imaging speed is evaluated by the number of frames per second. The time used for outputting one frame is the time from when the pulsed laser transmits the pulsed to the time when the FPGA outputs an image. The imaging speed of the proposed system is in the same orders of magnitude as that of the other systems because these effect factors on the imaging speed of the proposed system are the same order of magnitude as that of the other systems. The resolution of the proposed system may be insufficient to recognize the target. Therefore, if the users want to recognize the targets effectively, a higher resolution may be needed. However, increasing the resolution requires a larger number of the microlens array and the APD detector array, and this requirement raises the manufacturing difficulty and cost. Therefore, the users should select reasonable resolution depending on the practical application.

**Table 2:** Comparison of the proposed system and some current state of art flash Lidars.

System	Wavelength(nm)	FOV	Resolution	Imaging speed
OFILR^47^	1520~1580	2.55° × 2.55° (44.45 mrad × 44.45 mrad)	35 × 35	0.0289 fps
ASC FLVC^48^	1570	45°	128 × 128	10~60 fps
Gen-III^49^	532	0.59° × 0.59° (10.3 mrad × 10.3 mrad)	32 × 32	4.5 fps
Work of ref.^50^	850	6° × 6°	16 × 16	16 fps
GLidar-II^51^	905	1.2° × 1.2°	5 × 5	—
This work	1550	52° × 52°	30 × 18	Dozens of fps

## Conclusions

In this study, a pulsed-laser 3D imaging inspired by the compound and human hybrid eye is proposed. The principle of the proposed system is introduced and the mathematical model of the receiving system is developed. The receiving system has 18 × 30 (rings × sections) microlenses that are distributed on a curved surface to mimic the large FOV feature of the compound eye. Meanwhile, the location distribution of these microlens on the curved surface and the optical parameters of these microlens are non-uniform to mimic the space-variant resolution property of the human eye. Simulation experiments are carried out on the proposed system, including imaging with a large FOV and space-variant resolution as well as rotation and scaling invariances. The results show that the entire FOV of the proposed system is up to 52°, and the system has a high resolution in the center FOV and a low resolution in the peripheral FOV. The rotation and scaling invariances are verified, and the results indicate that the proposed system has these two invariances of the human eye. The SNR of the proposed system is analyzed, and the results show that the SNR increases with the increase in the number of rings and the maximum SNR locates at the outmost periphery area. The main purpose of this paper is to provide the modeling and the simulation verification of the proposed pulsed-laser 3D imaging system inspired by compound and human hybrid eye. Our future works include performing experimental validation to obtain the distance and the intensity images.
